# Parasite powerhouse: A review of the *Toxoplasma gondii* mitochondrion

**DOI:** 10.1111/jeu.12906

**Published:** 2022-05-04

**Authors:** Madelaine M. Usey, Diego Huet

**Affiliations:** ^1^ 308512 Department of Cellular Biology University of Georgia Athens Georgia USA; ^2^ 308512 Center for Tropical and Emerging Global Diseases University of Georgia Athens Georgia USA; ^3^ 308512 Department of Pharmaceutical and Biomedical Sciences University of Georgia Athens Georgia USA

**Keywords:** apicomplexan, biosynthesis, drug target, electron transport chain, metabolism, mitochondrial genome, mitochondrial protein import

## Abstract

*Toxoplasma gondii* is a member of the apicomplexan phylum, a group of single‐celled eukaryotic parasites that cause significant human morbidity and mortality around the world. *T*. *gondii* harbors two organelles of endosymbiotic origin: a non‐photosynthetic plastid, known as the apicoplast, and a single mitochondrion derived from the ancient engulfment of an α‐proteobacterium. Due to excitement surrounding the novelty of the apicoplast, the *T*. *gondii* mitochondrion was, to a certain extent, overlooked for about two decades. However, recent work has illustrated that the mitochondrion is an essential hub of apicomplexan‐specific biology. Development of novel techniques, such as cryo‐electron microscopy, complexome profiling, and next‐generation sequencing have led to a renaissance in mitochondrial studies. This review will cover what is currently known about key features of the *T*. *gondii* mitochondrion, ranging from its genome to protein import machinery and biochemical pathways. Particular focus will be given to mitochondrial features that diverge significantly from the mammalian host, along with discussion of this important organelle as a drug target.

Mitochondria are double membrane‐bound organelles that play central roles in energy production and cellular metabolism. The origin of mitochondria can be traced back to a significant evolutionary event that occurred around 1.5 billion years ago. One theory holds that the advent of mitochondria stemmed from a single endocytic event in which a proto‐eukaryote engulfed an α‐proteobacterium and retained it as an organelle (Archibald, 2015; Gray, 2012). However, there is an emerging hypothesis that mitochondria could have instead originated through a process of symbiosis between an α‐proteobacterium and an archaebacterium (Martin & Müller, 1998; Zachar & Szathmáry, 2017). Regardless of their origin, mitochondrial function can vary dramatically between different taxonomic lineages, likely as a result of ecological specializations that have occurred since the first eukaryote appeared. Surviving in various environments requires a high degree of metabolic adaptability, and the evolution of mitochondria and its derived organelles–such as mitosomes and hydrogenosomes, which are found in anaerobic organisms–likely allowed unicellular eukaryotes to accomplish this feat.

Apicomplexans are a group of intracellular parasites in the eukaryotic clade Myzozoa, which also includes dinoflagellates and chromerids. Importantly, apicomplexan parasites are the causative agents of major human diseases including malaria, toxoplasmosis and cryptosporidiosis (Cavalier‐Smith & Chao, 2004). While apicomplexans from the genus *Cryptosporidium* possess a mitosome, *Plasmodium* spp. and *Toxoplasma gondii* harbor a single mitochondrion (Mathur et al., 2021). In this review, we will discuss both canonical and divergent aspects of the *T*. *gondii* mitochondrion.

The life cycle of *T*. *gondii* is composed of an asexual and a sexual phase. During the asexual phase, tachyzoites–the fast‐replicating form of the parasite–invade host cells and replicate intracellularly. After several rounds of replication, they will egress from the host cell, destroying it in the process. Newly egressed tachyzoites will repeat this cycle of invasion, replication, and egress, termed the lytic cycle. Tachyzoites can also transition into bradyzoites, a slow‐replicating stage that forms tissue cysts in the host. If ingested by a felid, bradyzoites can differentiate into sexual stages (Attias et al., 2020). However, little is known about the mitochondrion in bradyzoites and the sexual stages of *T*. *gondii* and unless specified otherwise, we will refer to the mitochondrial biology of tachyzoites in this review.

Once inside a host cell, intracellular parasites harbor a mitochondrion that loops around the periphery of the cell in a lasso shape (Nishi et al., 2008; Ovciarikova et al., 2017). In extracellular parasites, however, the organelle rapidly adopts a sperm‐like or collapsed conformation (Figure 1), suggesting that the transition between extracellular and intracellular stages of *T*. *gondii* induces mitochondrial morphology changes (Ovciarikova et al., 2017). Interestingly, this change is reversible, as the mitochondrion of extracellular parasites adopts a lasso shape once they invade a new host cell (Ovciarikova et al., 2017). This observation suggests that an active mechanism could be responsible for the mitochondrial morphology changes observed during the *T*. *gondii* lytic cycle.

**FIGURE 1:**
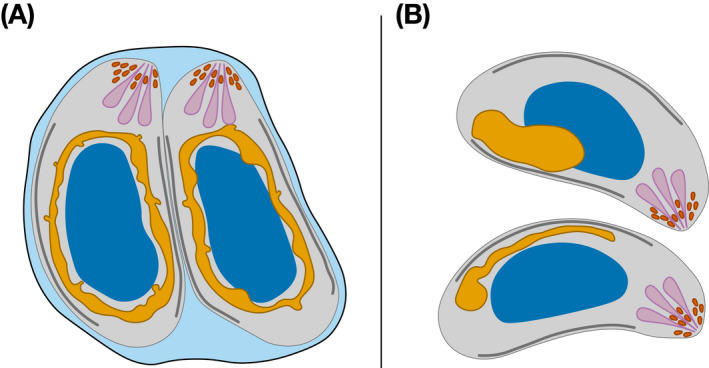
Mitochondrial morphology changes throughout the *Toxoplasma gondii* lytic cycle. During the transition from the host cell cytosol to the extracellular environment, the mitochondrion of *T*. *gondii* undergoes drastic morphologic changes. Intracellular *T*. *gondii* tachyzoites possess a lasso‐shaped mitochondrion spanning almost the whole length of the parasite (A). In contrast, extracellular parasites present collapsed (B, top) or sperm‐like (B, bottom) mitochondria. To orient the cells, the inner membrane complex (gray) and two organelles of the apical complex, rhoptries (pink) and micronemes (orange), are depicted

Historically, most functions of the *T*. *gondii* mitochondrion have been inferred by identifying genes with well‐studied homologs in other organisms, such as yeast and mammals. These comparative genomic studies have shown that the *T*. *gondii* mitochondrion possesses many of the canonical functions found in other eukaryotes, such as a functional tricarboxylic acid (TCA) cycle along with an electron transport chain (ETC) that enables oxidative phosphorylation (OXPHOS), and biosynthetic pathways such as the iron‐sulfur cluster, heme, and pyrimidine synthetic pathways (Hortua et al., 2016; MacRae et al., 2012; Seeber et al., 2008; Vercesi et al., 1998). Numerous mitochondrial housekeeping functions present in the last eukaryotic common ancestor, including a protein import system along with transcription and translation machinery for the mitochondrial genome, are also present in *T*. *gondii*. Another key ultrastructural component of mitochondria is their cristae, which are folds of the inner mitochondrial membrane (IMM) that provide a framework for the protein complexes involved in cellular energy production. Whereas most yeast and mammalian cells harbor mitochondria with lamellar cristae (Pánek et al., 2020), the *T*. *gondii* mitochondrion contains unique bulbous cristae, which will be discussed in more detail later (Muhleip et al., 2021).

Although the mitochondrion of *T*. *gondii* hosts many canonical functions, it is also a divergent organelle when compared to other well‐studied eukaryotic models. Two recent studies identified approximately 400 mitochondrial proteins, with half of them lacking any homologs in humans and yeast (Barylyuk et al., 2020; Seidi et al., 2018). In fact, compared to other subcellular structures, the mitochondrion has the highest number of proteins essential for parasite survival that are also exclusively conserved in apicomplexans (Indispensable Conserved Apicomplexan Proteins, or ICAPs) (Sidik et al., 2016), demonstrating that this organelle hosts numerous important and specific aspects of apicomplexan biology (Figure 2). Elucidating the function of these ICAPs will undoubtedly yield insight into the unique mitochondrial biology of apicomplexans. Moreover, since these proteins are absent from the host, they could serve as attractive drug targets.

**FIGURE 2:**
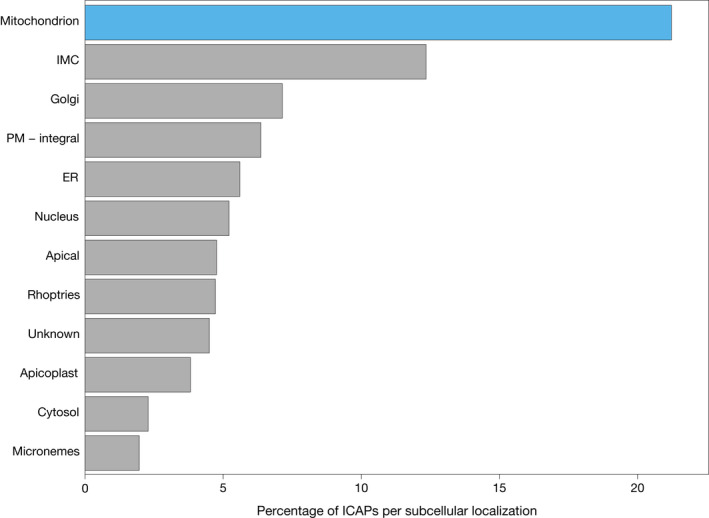
Percentage of indispensable conserved apicomplexan proteins (ICAPs) per subcellular localization. Proportions of ICAPs in every subcellular localization as defined by the spatial proteome of *Toxoplasma gondii* (Barylyuk et al., 2020). Of all the proteins localized to the mitochondrion, 21% are ICAPs, a number significantly higher compared to other subcellular compartments

Whereas the focus of this review is the mitochondrion of *T*. *gondii*, work on *P*. *falciparum* has provided a framework for studying this organelle, and this will be mentioned throughout the review when applicable.

## DECEPTIVE DNA: THE HUNT FOR DETAILS ON THE MITOCHONDRIAL GENOME

Mitochondria contain their own genome, which is a remnant of their bacterial origin. The first mitochondrial genomes from apicomplexans were identified in two *Plasmodium* species (Aldritt et al., 1989; Vaidya et al., 1989). Unexpectedly, these genomes are remarkably small: about 6 kb, whereas the human mitochondrial genome is 16 kb in length. Sequencing demonstrated that these reduced mitochondrial genomes only contain three cytochrome protein‐coding genes from the ETC (cytochrome c oxidase subunit I, *coxI*; cytochrome c oxidase subunit III, *coxIII*; and cytochrome b, *cob*) along with fragmented mitochondrial ribosomal RNA (rRNA) genes. To this day, all sequenced apicomplexan mitochondrial genomes have the same size, but the genome architecture along with the gene orientation vary across the phylum (Hikosaka et al., 2010, 2013). Dinoflagellates, a sister group to apicomplexans, also have an extremely reduced mitochondrial genome consisting of fragmented rRNA genes and the same three cytochrome genes, but the fragmentation seems to have evolved independently (Gagat et al., 2017).

**FIGURE 3:**
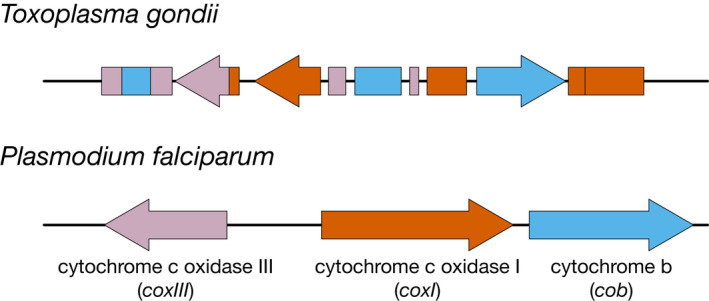
Mitochondrial DNA organization in *Toxoplasma gondii* and *Plasmodium falciparum*. In *T*. *gondii*, the coding sequences of ribosomal RNA and cytochrome (fragmented arrows) genes are fragmented and scrambled, and non‐random arrangements of multiple sequence blocks allow for the transcription of full‐length genes. In contrast, the mitochondrial DNA of *P*. *falciparum* contains the full coding sequence of all three cytochrome protein‐coding genes. For clarity, the fragmented ribosomal RNA and other RNA genes, present in all apicomplexan mitochondrial DNAs, are not represented

Until very recently, most of what we knew about the mitochondrial genome in *T*. *gondii* was inferred from work done in *Plasmodium*, as numerous attempts to sequence the mitochondrial DNA (mtDNA) of *T*.* gondii* were unsuccessful. This was likely due to the presence of nuclear‐encoded sequence fragments of mitochondrial origin (NUMTs) in the *T*.* gondii* nuclear genome. NUMTs are fragments of mtDNA that integrate into the nuclear genome following a DNA double‐strand break. These sequences can interfere with mtDNA isolation, amplification and in silico genome assembly approaches. The unprecedented number of NUMTs (over 9000) found in *T*. *gondii* consequently made any attempt to characterize the mtDNA of this parasite a daunting task (Namasivayam & Kissinger, 2015). Excitingly, recent advances in second‐ and third‐generation sequencing techniques finally allowed for determination of the sequence and structure of the mtDNA in *T*. *gondii* (Namasivayam et al., 2021). This work revealed that the mtDNA is 5.9 kb long and consists of 21 sequence blocks (SBs), named from A to V. Ranging from 40 to 1050 base pairs, all SBs are actively transcribed and encode proteins or rRNA. Remarkably, none of these SBs encodes for a full‐length protein or rRNA (Figure 3). Rather, sequencing reads showed that non‐random arrangements of multiple SBs encode full‐length cytochrome and rRNA genes (Namasivayam et al., 2021). For example, blocks E, A and T encode *cob*, whereas blocks V, L, J, B and M encode *coxIII*. Homoplasmy of the SBs is maintained by an unknown mechanism, but could be explained by the presence of TgMSH1, a protein previously identified as a yeast MutS homolog that is involved in DNA mismatch repair and in maintaining mtDNA stability, (Garbuz & Arrizabalaga, 2017) or by mtDNA intermolecular recombination.

Although the sequence of the mtDNA in *T*. *gondii* was finally solved, the exact topology of the genome and the mechanisms enabling its replication are still unknown. On one hand, the SB arrangement and orientation suggests that the mtDNA organizes into multiple chromosomes, in a conformation reminiscent of the mitochondria of dinoflagellates (Flegontov et al., 2015). In other apicomplexans, on the other hand, mtDNA is contained within a single chromosome (Namasivayam et al., 2021). Advances in mitochondrial purification and mtDNA sequencing from single organelles could help in addressing these remaining questions on mtDNA topology and replication.

In *T*. *gondii*, only a few studies have addressed mitochondrial genome transcription and translation, but we know that both processes are active in this parasite. To translate the three protein‐encoding genes encoded within its mtDNA, the parasite must import a complete set of transfer RNAs (tRNAs) from the cytosol, since the mtDNA of *T*. *gondii* does not encode any tRNA genes (Esseiva et al., 2004; Namasivayam et al., 2021). Predicted elongation factors and ribosomal proteins localize to the mitochondrion, and six of the SBs found in the mtDNA encode fragments of the large and small ribosomal subunits of the mitochondrial ribosome, also known as the mitoribosome (Lacombe et al., 2019; Namasivayam et al., 2021; Pino et al., 2010; Seidi et al., 2018).

Although it is assumed that the mitoribosome drives the synthesis of the three proteins encoded in the mtDNA of *T*. *gondii*, the complete structure and composition of the complex remains elusive. Work in *P*.* falciparum* and *T*. *gondii* has identified some subunits of the complex. In *P*. *falciparum*, depletion of the putative mitoribosomal subunit L13 results in the loss of mitochondrial membrane potential, ETC deficiency, and aberrant mitochondrial morphology (Ke et al., 2018). Further work in *T*. *gondii* showed that under native conditions, three putative mitoribosome subunits, termed TgbL12m, TguL3m, and TgmS35, migrate similarly at approximately 1000 kDa: a molecular weight that could correspond to the mitoribosomal complex (Lacombe et al., 2019). It is worth mentioning that work on the structure of the mitoribosome of *Chlamydomonas reinhardtii*, an organism with fragmented mitochondrial rRNAs, estimated the molecular weight of the complex to approximately 2400 kDa (Waltz et al., 2021). Consequently, the 1000 kDa complex observed by Lacombe et al. could correspond to a mitoribosomal assembly intermediate. Lacombe and colleagues also demonstrated that conditional depletion of TgmS35 impaired parasite growth and reduced ETC complex IV activity. Since complex IV contains two mitochondrially encoded subunits (COXI and COXIII), these results suggest that TgmS35 depletion reduces translation of COX1 and COX3 mRNAs from the mitochondrial genome (Lacombe et al., 2019). Surprisingly, complexome profiling of *P*. *falciparum* and *T*.* gondii* mitochondria identified the ETC and ATP synthase complexes of these parasites but failed to detect a mitoribosome (Evers et al., 2021; Maclean et al., 2021). Further work is, therefore, necessary to establish the structure and subunit composition of the apicomplexan mitoribosome.

## TRANSLOCATE, FOLD, REPEAT: MITOCHONDRIAL PROTEIN IMPORT

As most mitochondrial proteins are nucleus‐encoded, they must be targeted and transported into the mitochondrion following translation. In many model organisms, a major player in mitochondrial protein import is the large multisubunit TOM (translocase of the outer membrane) complex, which facilitates protein transport from the cytoplasm across the outer mitochondrial membrane (OMM) (Brix et al., 1997). Within this complex, the β‐barrel protein Tom40 is an integral membrane protein that forms a hydrophilic pore to allow for protein import (Hill et al., 1998). Another key component of the TOM complex is Tom22, which has a single transmembrane domain critical for stable higher‐order assembly of the TOM complex. Its cytosolic domain binds proteins that are to be imported and serves as a docking point for two other receptor subunits, Tom20 and Tom70 (van Wilpe et al., 1999). The portion of Tom22 that extends into the intermembrane space (IMS) binds proteins as they enter the mitochondrion, assisting in their translocation through the TOM complex pore (Moczko et al., 1997). Other small Tom proteins also play roles in the complex. Tom5 assists in protein entry into the Tom40 pore, while Tom6 and Tom7 play structural roles (Brix et al., 1997). Using comparative genomic approaches, a study in *T*. *gondii* was able to identify homologs of Tom40 and Tom22 (TgTom40 and TgTom22). Notably, homologs of Tom20, Tom70, or Tom5 could not be identified (van Dooren et al., 2016). While initial gene homology studies failed to identify a Tom7 homolog, alignment of Tom7 sequences from several organisms revealed a conserved motif in the single transmembrane domain that ultimately led to identification of TgTom7. Further characterization showed that both TgTom22 and TgTom7 are important for tachyzoite growth and are part of a ~400 kDa complex with TgTom40. Additionally, both proteins were confirmed to play roles in *T*.* gondii* TOM complex stability and mitochondrial protein import (van Dooren et al., 2016).

Once proteins have been imported into the IMS via the TOM complex, many will interact with a translocase of the inner membrane (TIM) complex to get to their final destination. Specifically, for carrier proteins on their way to the IMM, the TIM22 complex serves as a chaperone (Jensen & Dunn, 2002). OMM β‐barrel carrier proteins, responsible for protein, lipid, and metabolite import into the organelle, first interact with small TIM chaperones, then are transferred to the sorting and assembly machinery (SAM), which folds and inserts them into the OMM. The SAM complex consists of a central β‐barrel protein, Sam50, and two accessory proteins, Sam35 and Sam37 (Diederichs et al., 2020). While comparative genomics identified *T*.* gondii* homologs of Sam50 and Tim22 that were localized to the mitochondrion (Sheiner et al., 2015), other components of the TIM22 and SAM complexes remain to be identified (van Dooren et al., 2016).

Proteins destined for the matrix require additional chaperones to mediate their transport across the IMM. In many model organisms, this transport process is directed by the presence of a mitochondrial targeting signal (MTS): an N‐terminal domain with an overall positive charge and amphipathic α‐helices (von Heijne et al., 1989). Indeed, approximately 60% of the *T*. *gondii* mitochondrial matrix proteins are predicted to harbor a canonical MTS (Seidi et al., 2018). This could illustrate either that many *T*. *gondii* matrix proteins lack canonical targeting sequences or that there is a high percentage of false positives in the matrix proteome (Seidi et al., 2018).

The transport of matrix proteins to their final location relies on the TIM23 complex. Within this complex, integral membrane proteins Tim23 and Tim17 function as the channel for protein transport, and the Tim50 subunit plays an important role in transporting proteins from the TOM complex to the TIM23 complex (Jensen & Dunn, 2002; Mokranjac et al., 2003). While protein import into the matrix is initially driven by the mitochondrial membrane potential, complete translocation is an active process that relies on the use of a motor. The core of the motor consists of Hsp70, which interacts directly with the preprotein being translocated. Tim44 is a peripheral membrane protein, which tethers Hsp70 to the TIM23 channel and Pam18 is a J‐protein that stimulates the ATPase activity of Hsp70 (D’Silva et al., 2008). Homologs of Tim23, Tim17, Tim50, Tim44, Hsp70 and Pam18 were identified in the *T*. *gondii* genome and epitope tagging validated that Tim23, Tim50, and Pam18 homologs localize to the parasite mitochondrion (van Dooren et al., 2016).

After proteins have been transported across the IMM and into the matrix, their MTS must be cleaved. This function is carried out by the mitochondrial processing peptidase (MPP) (Glaser et al., 1994). MPP is a heterodimeric enzyme consisting of an MPP*α* subunit, which binds precursor proteins, and an MPP*β* subunit, which contains the active site that catalyzes cleavage of the MTS (Luciano et al., 1997). While MPP is soluble in the matrix of yeast and mammalian mitochondria (Kleiber et al., 1990; Yang et al., 1988), it is part of ETC complex III in plants (Eriksson et al., 1996). A homolog of MPP*α* was initially localized to the *T*. *gondii* mitochondrion, and later studies confirmed that as in plants, TgMPP*α* exists as part of a complex containing other canonical complex III subunits, including MPP*β* (van Dooren et al., 2016; Hayward et al., 2021; Maclean et al., 2021).

Another important class of proteins imported into the mitochondrion reside in the IMS. Most IMS‐resident proteins contain cysteine residues in separate *α*‐helices that are generally separated by either three or nine amino acids (Modjtahedi et al., 2016). Consequently, these proteins are often termed twin CX3C and twin CX9C proteins (Herrmann & Riemer, 2012). Instead of an N‐terminal MTS, most IMS proteins possess an internal targeting sequence termed the IMS‐targeting sequence (ITS). This signal sequence is characterized by a groove containing hydrophobic and aromatic residues that permit interaction with a chaperone following their import through the TOM complex (Herrmann & Riemer, 2012). Cysteines of the IMS proteins are oxidized to form disulfide bonds by the chaperone Mia40, which is then reoxidized by its partner Erv1, as they are known in yeast (Herrmann & Riemer, 2012). Interestingly, homology‐based searches fail to identify a Mia40 homolog in the *T*. *gondii* genome (van Dooren et al., 2016). However, twin CX9C proteins have been identified in *T*. *gondii*. Specifically, three apicomplexan‐specific subunits in the *T*. *gondii* ATP synthase possess CX9C domains, and two were predicted to be essential for parasite survival (Huet et al., 2018; Muhleip et al., 2021; Sidik et al., 2016). Previous studies have shown that other protozoa, including trypanosomatids, similarly express twin cysteine proteins but seem to lack a Mia40 homolog. This apparent absence could be explained by sequence divergence that prevents identification of Mia40 homologs. It has also been hypothesized that early eukaryotes may have solely utilized Erv1 for twin cysteine protein import and that evolution of Mia40 represents a streamlined version of this pathway (Allen et al., 2008). Interestingly, sequence homology studies have identified three Erv1 homologs in the *T*. *gondii* genome (van Dooren et al., 2016). However, protozoan Erv1 was unable to complement yeast lacking Mia40 (Specht et al., 2018). As some twin cysteine IMS‐resident proteins are essential in *T*. *gondii*, future studies of their potentially divergent import machinery could provide both functional and evolutionary insights.

While most mitochondrial proteins are encoded in the nuclear genome then transported into the mitochondrion by the aforementioned pathways, the three mtDNA‐encoded subunits of the *T*. *gondii* ETC must also be inserted into the IMM. In other eukaryotes, this process is mediated by Oxa1, which associates with mitoribosomes to mediate co‐translational insertion of mtDNA‐encoded ETC subunits into the IMM (Hell et al., 2001; Keil et al., 2012). Oxa1 has also been shown to play a role in the import and biogenesis of IMM carrier proteins (Hildenbeutel et al., 2012). Recently, a homolog of Oxa1 was found in the *T*. *gondii* genome (van Dooren et al., 2016). Though some putative mitoribosomal subunits have been identified in *T*. *gondii* as discussed previously (Lacombe et al., 2019), future studies of the *T*. *gondii* Oxa1 homolog could provide insight into this elusive complex. In summary, while many key components of various mitochondrial import systems have been identified in *T*. *gondii*, further characterization of these systems, and identification of missing accessory subunits, remains to be completed. It is also important to note that while this section focused heavily on translocation and import systems conserved in many model organisms, the systems used by *T*. *gondii* may diverge significantly.

## COLOSSAL COMPLEXES OF THE ELECTRON TRANSPORT CHAIN

A key component of the mitochondrion is the ETC, a series of protein complexes located in the IMM. ETC complexes transfer electrons derived from metabolic substrates, ultimately reducing molecular oxygen to form water and pumping protons across the IMM into the IMS (Mansilla et al., 2018). This process generates a proton gradient and an electrical potential across the IMM, which together form the mitochondrial membrane potential. This potential can then be harvested by the ATP synthase to catalyze the conversion of ADP to ATP through OXPHOS (Rathore et al., 2019; Zorova et al., 2018). While the following section will delve deeper into mitochondrial metabolism and biosynthetic pathways, this section will focus on the protein composition of the ETC. Interestingly, recent studies have highlighted aspects of the *T*. *gondii* ETC that diverge significantly from the canonical ETC found in many other eukaryotes. Specifically, the dehydrogenases that deliver electrons to the ETC, as well as the size and subunit composition of ETC complexes, differ considerably in *T*. *gondii* and other apicomplexans.

Electrons are delivered to the ETC through oxidation of metabolites. These reactions are catalyzed by various dehydrogenases and result in reduction of the mobile electron carrier coenzyme Q (CoQ) (Hayward & van Dooren, 2019). In mammals, four dehydrogenases deliver electrons to CoQ: dihydroorotate dehydrogenase (DHODH), glycerol‐3‐phosphate dehydrogenase (G3PDH), complex I (NADH:ubiquinone dehydrogenase), and complex II (succinate dehydrogenase). The *T*. *gondii* homolog of DHODH is essential for the lytic cycle and plays a critical role in the de novo synthesis of pyrimidines by catalyzing the oxidation of dihydroorotate into orotate (Hortua et al., 2012; Sidik et al., 2016). On the other hand, G3PDH has not been extensively characterized in *T*. *gondii*. In mammalian mitochondria, G3PDH catalyzes the oxidation of glycerol‐3‐phosphate (G3P) into dihydroxyacetone phosphate (DHAP) (Mráček et al., 2013). G3PDH is not essential and an early biochemical study of *T*.* gondii* illustrated that supplying exogenous G3P as the only metabolic substrate did not induce OXPHOS in permeabilized tachyzoites (Sidik et al., 2016; Vercesi et al., 1998). However, a later study illustrated that G3P supplementation restored depolarized mitochondrial membrane potential when other ETC dehydrogenases were chemically inhibited (Lin et al., 2009). Thus, contributions of G3PDH to the *T*. *gondii* ETC remain unclear. Interestingly, *T*. *gondii* possesses another dehydrogenase not found in its mammalian host: the malate:quinone oxidoreductase (MQO). MQO, found in many bacteria and single‐celled eukaryotes, catalyzes the conversion of malate to oxaloacetate as part of the TCA cycle. While MQO catalyzes this conversion in an irreversible reaction and reduces CoQ, the canonical mammalian malate dehydrogenase (MDH) catalyzes the same reaction reversibly and ultimately reduces NAD+ (Acharjee et al., 2021). *T*.* gondii* also expresses MDH and localization studies found that MQO and MDH both localize to the mitochondrion (Fleige et al., 2008). As studies in other systems have illustrated that conversion of malate to oxaloacetate by MQO is energetically favored compared to the reaction catalyzed by MDH, it has been hypothesized that these two enzymes may work to catalyze reactions in opposite directions (Molenaar et al., 2000). However, this remains to be determined in *T*. *gondii*.

Another divergent feature of the *T*. *gondii* ETC is exemplified by the complex that accepts electrons from NADH. In mammals, this is carried out by a ~1‐MDa type I NADH:ubiquinone dehydrogenase (complex I, NDH1), which oxidizes NADH and reduces CoQ (Blaza et al., 2017; Vinothkumar et al., 2014). Initial reports showed that treatment with the complex I inhibitor rotenone did not affect mitochondrial oxygen consumption in *T*. *gondii* (Vercesi et al., 1998), indicating lack of complex I activity. It was later discovered that *T*. *gondii* expresses two non‐redundant isoforms of type II NADH dehydrogenases (NDH2), which are not found in mammalian cells (Hayward & van Dooren, 2019; Lin, Gross, et al., 2011). NDH2 enzymes are a single subunit, making them significantly smaller than multisubunit NDH1 enzymes. Additionally, unlike NDH1, NDH2 proteins do not pump protons across the IMM (Blaza et al., 2017). Both *T*. *gondii* NDH2 isoforms play roles in maintaining normal mitochondrial membrane potential and proper parasite growth through their delivery of electrons to the ETC (Lin, Gross, et al., 2011). This loss of complex I and replacement with NDH2 predates the evolution of apicomplexans from their free‐living myxozoan relatives (Flegontov et al., 2015). As complex I is a major source of reactive oxygen species (ROS), this replacement may have reduced the production of damaging ROS and facilitated the adaptation to intracellular parasitic life (Fisher et al., 2007).

As another entry point for electrons into the ETC, complex II plays a critical role in the TCA cycle by catalyzing the oxidation of succinate to fumarate coupled with reduction of CoQ (Sun et al., 2005). In mammals and yeast, this complex is approximately 130 kDa and consists of four subunits (Schägger & Pfeiffer, 2000; Sun et al., 2005). Homology searches revealed that genes encoding the soluble SDHA and SDHB subunits can be identified in the *T*. *gondii* genome, while the other two integral membrane subunits allowing for electron transfer to CoQ have remained unidentified (Maclean et al., 2021). A recent *T*. *gondii* mitochondrial complexome study provided the first investigation into the composition of the apicomplexan complex II. This study found that SDHB migrated as part of a ~500 kDa complex along with seven other co‐migrating proteins all annotated as “hypothetical.” To determine whether co‐migrating proteins could represent complex II subunits, the authors utilized phylogenetic homology searches (Maclean et al., 2021). Interestingly, different species of the apicomplexan *Cryptosporidium* possess ETCs that have been truncated to varying degrees (Hayward & van Dooren, 2019). Since complex II is found in *Cryptosporidium muris*, but not in *Cryptosporidium parvum*, it is likely that *T*. *gondii* complex II subunits will share the same phylogenetic distribution. Indeed, four of the seven co‐migrating proteins are conserved in *Cryptosporidium muris* but not in *Cryptosporidium parvum* (Maclean et al., 2021), providing additional support for their assignment as bona fide complex II subunits. Nonetheless, the *T*. *gondii* complex II is much larger in size than mammalian and yeast complexes and its putative novel subunits warrant additional study.

Multiple recent studies have also characterized *T*. *gondii* complex III. In most model organisms, complex III consists of 10 subunits with three main functional groups: the Rieske center, which contains a Fe‐S cluster, along with two heme‐containing cytochromes (b and c1). In two successive cycles, two reduced CoQ molecules are oxidized at the Q_0_ site of complex III and their electrons are transferred to two cytochrome c molecules. During this process, one oxidized CoQ molecule is reduced to QH2 at the Q_i_ site and four protons are pumped into the IMS (Crofts, 2004). Homology searches in *T*. *gondii* revealed genes encoding six canonical subunits, including Rieske and cytochrome c1 (Maclean et al., 2021). Surprisingly, the gene encoding cytochrome c1 also encodes a subunit of the *T*. *gondii* ATP synthase. Homology searches indicate that residues 179–331 contain a highly conserved domain found in cytochrome c1 proteins, while residues 32–153 are found within the ATP synthase structure (Maclean et al., 2021; Muhleip et al., 2021). It is not currently known whether this gene encodes two separate proteins or whether the protein is cleaved upon import into the mitochondrion. In yeast and bovine cells, complex III exists as a homodimer of approximately 500 kDa in mass (Iwata et al., 1998; Schägger & Pfeiffer, 2000). Two separate studies in *T*. *gondii* determined the protein composition of complex III, independently identifying the same set of 11 subunits as part of a ~675 kDa complex. This complex includes the six canonical nuclear‐encoded subunits, the mtDNA‐encoded cytochrome b, and four novel subunits (Hayward et al., 2021; Maclean et al., 2021). Two novel subunits display homology to known bovine and yeast complex III subunits (QCR8 and QCR9), one appears to be conserved among myxozoans (QCR11), and another seems to be apicomplexan‐specific (QCR12) (Hayward et al., 2021; Maclean et al., 2021). All four novel subunits were shown to play important roles in maintaining mitochondrial membrane potential, complex III structure, and parasite fitness (Maclean et al., 2021; Sidik et al., 2016). Intriguingly, these studies of complex III provided the first evidence that respiratory supercomplexes, which increase ETC efficiency by allowing direct substrate channeling between complexes, may form in *T*. *gondii* as they do in other eukaryotes (Genova & Lenaz, 2014; Maclean et al., 2021).

Many groups have also recently worked to elucidate the composition of *T*. *gondii* complex IV. Complex IV transfers electrons from the reduced cytochrome c molecules generated by complex III to the final electron acceptor, oxygen, and pumps protons from the matrix into the IMS (Mansilla et al., 2018). A 2018 study of the *T*. *gondii* mitochondrial proteome provided early functional data on one subunit of complex IV termed ApiCox25 (Seidi et al., 2018). This fitness‐conferring subunit was determined to be critical for mitochondrial oxygen consumption and was found as part of a ~600‐kDa complex, in which it plays important structural and stability roles. Immunoprecipitation studies revealed ApiCox25 associated both with homologs of canonical complex IV subunits and with novel, myzozoan‐specific putative subunits (Seidi et al., 2018). Despite difficulties in detecting the mtDNA‐encoded COXI subunit by some studies (Seidi et al., 2018), 16 subunits have been confirmed as part of *T*. *gondii* complex IV (Lacombe et al., 2019; Maclean et al., 2021; Seidi et al., 2018). Mass calibration experiments revealed that complex IV subunits migrate as part of a 460 kDa complex (Maclean et al., 2021), instead of a 600‐kDa complex as previously reported (Seidi et al., 2018). Discrepancies between the observations by Maclean et al. (2021) and Seidi et al. (2018) may be due to recently uncovered inaccuracies when using commercially available protein ladders, which consist of soluble complexes, to estimate sizes of large membrane‐bound ETC complexes via native gel electrophoresis (Evers et al., 2021). Since the predicted mass of all known 16 complex IV subunits is only 364 kDa, significantly lower than 460 kDa, additional proteins may be members of complex IV. Indeed, six other co‐migrating proteins, mostly specific to apicomplexans, were observed, bringing the total mass up to 438 kDa and much closer to the 460 kDa estimate (Maclean et al., 2021). Thus, the *T*. *gondii* complex is more than twice as large as yeast and bovine complex IV, which are approximately 200 kDa (Schägger & Pfeiffer, 2000; Tsukihara et al., 1996). All six putative novel subunits are predicted to localize to mitochondrial membranes, and two previous datasets of complex IV subunits revealed that these six subunits were identified, but not assigned to complex IV (Lacombe et al., 2019; Seidi et al., 2018). As these six putative subunits are not found in the mammalian host, further characterization of their roles and functions is needed.

**FIGURE 4:**
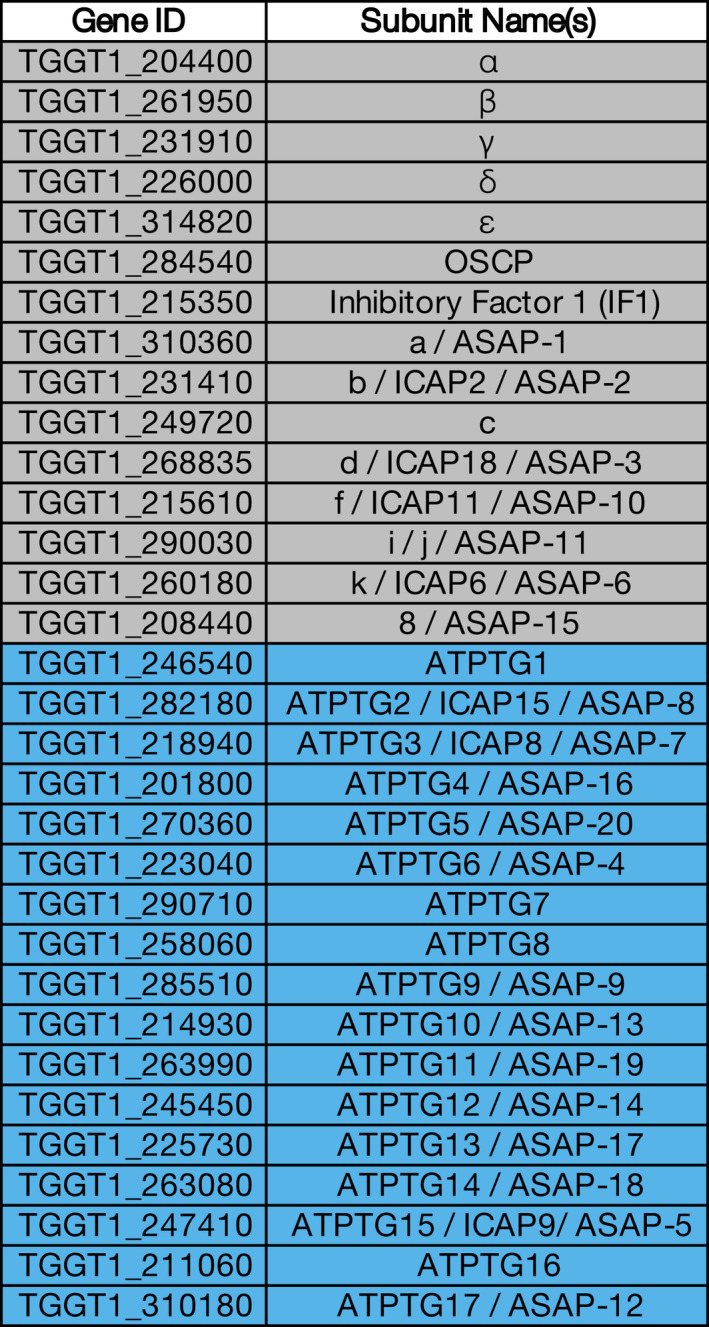
*Toxoplasma gondii* ATP synthase subunits. This table summarizes the subunits of the *T*.* gondii* ATP synthase as determined by Huet et al. (2018), Salunke et al. (2018), Muhleip et al. (2021), and Maclean et al. (2021). The rows highlighted in gray represent subunits conserved in mammals and yeast (Lau et al., 2008; Spikes et al., 2020), while the rows in blue represent phylum‐specific subunits

The proton gradient generated by the aforementioned ETC complexes can be harvested by the ATP synthase to catalyze the production of ATP. As protons flow down their electrochemical gradient and into the matrix via the membrane‐bound portion of the ATP synthase, the electrochemical energy is converted into rotational energy. At the membrane‐extrinsic catalytic sites, mechanical energy from this rotation is then converted to form a chemical bond between ADP and inorganic phosphate, resulting in production of ATP (Senior et al., 2002). While early metabolic studies demonstrated that *T*. *gondii* tachyzoites have a functional TCA cycle and generate ATP via OXPHOS (MacRae et al., 2012; Vercesi et al., 1998), homology searches failed to identify canonical subunits of the ATP synthase stator. This critical portion of the ATP synthase is required to counteract the rotational torque of the enzyme (Weber, 2007). A major breakthrough occurred in 2018 when two separate studies utilized immunoprecipitation and secondary structure predictions to identify structural homologs of canonical *a*, *b* and *d* subunits along with putative, phylum‐specific novel subunits (Huet et al., 2018; Salunke et al., 2018). A recently published cryo‐electron microscopy structure of the *T*.* gondii* ATP synthase confirmed these findings, ultimately demonstrating that the structure consists of 32 subunits, of which 15 are canonical and 17 are divergent and conserved only among apicomplexans or myzozoans (Figure 4) (Muhleip et al., 2021). Unexpectedly, many canonical subunits also contain apicomplexan‐specific extensions not identified outside the phylum (Muhleip et al., 2021). In summary, the *T*. *gondii* ATP synthase has nearly two times the number of subunits found in mammalian and yeast ATP synthase complexes (Lau et al., 2008; Spikes et al., 2020). Further, two *T*. *gondii* ATP synthase monomers are also capable of assembling into dimers with a total mass of 1860 kDa (Maclean et al., 2021; Muhleip et al., 2021), far exceeding the mass of ~1200‐kDa mammalian and yeast dimers (Lau et al., 2008; Walker et al., 1991). The small angle between *T*. *gondii* monomers in a dimer, 19°, differs considerably from the observed 100° dimer angle in mammalian mitochondria (Muhleip et al., 2021). The large ATP synthase dimer angle in mammals has been shown to induce membrane curvature and shape lamellar mitochondrial cristae (Blum et al., 2019). Interestingly, three *T*.* gondii* dimers can assemble into hexamers that can then form pentagonal pyramid structures not observed in other organisms. When a phylum‐specific subunit, ATPTG11, is knocked out, both the pentagonal pyramids and unique bulbous cristae morphology are disrupted, suggesting that this oligomeric structure contributes to proper cristae formation in *T*. *gondii* (Muhleip et al., 2021). Thus, the mechanism of cristae morphology maintenance seems to diverge significantly between *T*. *gondii* and mammals. As the *T*. *gondii* ATP synthase is essential for the tachyzoite form (Huet et al., 2018), its phylum‐specific subunits offer promising areas for future research. However, as three studies have provided different names for divergent *T*. *gondii* ATP synthase subunits, an agreement on a unified naming scheme would streamline future discussions.

**FIGURE 5:**
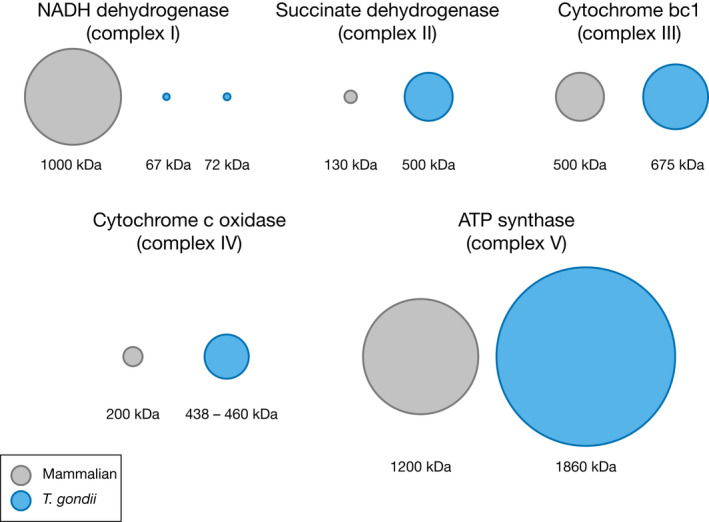
Size comparison of *Toxoplasma gondii* and mammalian ETC complexes. Each circle represents the scaled size in kDa of each ETC complex in gray (mammals) or *T*. *gondii* (blue). Note that *T*. *gondii* does not possess a canonical complex I as in mammals (Vinothkumar et al., 2014), but instead possesses two isoforms of type II NADH dehydrogenases (NDH2) (Lin, Gross, et al., 2011). The sizes in kDa for the complexes were obtained from the following sources: complex I (Lin, Gross, et al., 2011; Vinothkumar et al., 2014) and ToxoDB, (https://toxodb.org/toxo/app), complex II (Maclean et al., 2021; Sun et al., 2005), complex III (Hayward et al., 2021; Iwata et al., 1998; Maclean et al., 2021), complex IV (Maclean et al., 2021; Tsukihara et al., 1996), complex V (Maclean et al., 2021; Walker et al., 1991)

In summary, *T*. *gondii* ETC and ATP synthase complexes are substantially larger than those in other eukaryotes (Figure 5). In addition, because the ETC plays an essential role in most apicomplexans (Hayward & van Dooren, 2019), functional characterization of phylum‐specific novel subunits could yield insights into both mitochondrial evolution and drug development against these pathogens.

## BIOSYNTHESIS AND BIOCHEMISTRY AND METABOLISM, OH MY!

As the powerhouse of the cell, the *T*. *gondii* mitochondrion hosts a wide array of biochemical and biosynthetic pathways. To power these pathways, the parasite can scavenge host metabolites through the parasitophorous vacuole membrane (PVM) (Martin et al., 2007; Schwab et al., 1994). A seminal study in 1998 first illustrated that the *T*. *gondii* mitochondrion possesses an active ETC and utilizes OXPHOS for energy production (Vercesi et al., 1998). However, later studies found that lactate fermentation was critical for parasite growth and suggested that ATP from OXPHOS was not critical for motility (Al‐Anouti et al., 2004; Pomel et al., 2008). Confusion about the importance of OXPHOS in *T*. *gondii* continued when it was discovered that the *T*.* gondii* homolog of pyruvate dehydrogenase (PDH) localized to the apicoplast. As this enzyme is necessary to convert pyruvate–produced via cytosolic glycolysis–into acetyl‐CoA to feed the mitochondrial TCA cycle, it became unclear how the parasites could generate acetyl‐CoA to power OXPHOS (Fleige et al., 2007). Nonetheless, *T*. *gondii* encodes homologs of all TCA cycle enzymes and subsequent studies showed that both glucose and glutamine are catabolized in their mitochondrion via a canonical TCA cycle (Blume et al., 2009; Fleige et al., 2008; MacRae et al., 2012). The TCA cycle was shown to be active in both intracellular and extracellular tachyzoites and its inhibition resulted in disruption of the lytic cycle and extracellular motility (MacRae et al., 2012). Interestingly, ^13^C labeling experiments estimated that more than 80% of ATP in extracellular parasites is produced by OXPHOS (MacRae et al., 2012). As extracellular tachyzoites are motile and infective within an hour of egress even without access to exogenous carbon sources, they must utilize an energy reserve system (Lin, Blume, et al., 2011). Indeed, *T*.* gondii* encodes enzymes involved in a gamma‐aminobutyric acid (GABA) shunt, which allows glutamate and glutamine to fuel the TCA cycle via GABA (MacRae et al., 2012). Both intracellular and extracellular tachyzoites maintain high levels of GABA, but these levels are rapidly depleted when extracellular parasites are deprived of exogenous carbon. Further, parasites lacking the first enzyme in the GABA shunt, glutamate decarboxylase (TgGAD), were less fit compared to wild‐type parasites (MacRae et al., 2012). The use of this GABA shunt as an extracellular energy reserve illustrates the metabolic malleability of *T*. *gondii* throughout its life cycle as it adapts to different environments.

Despite work in *T*. *gondii* illustrating the importance of the TCA cycle, the question of acetyl‐CoA production remained unanswered due to the localization of PDH to the apicoplast. However, studies in other organisms have illustrated that another enzyme, branched‐chain α‐ketoacid dehydrogenase (BCKDH), shares significant structural similarities and regulatory mechanisms with PDH (Zhang et al., 1989). Both enzymes catalyze similar reactions in which an initial α‐ketoacid is decarboxylated by the catalytic E1 subunit of the complex (Oppenheim et al., 2014). In mammalian cells, BCKDH degrades branched‐chain α‐ketoacids derived from the branched‐chain amino acids (BCAAs) leucine, isoleucine and valine, ultimately resulting in production of acetyl‐CoA (Kato et al., 2006). The *T*. *gondii* genome encodes all subunits of the BCKDH complex, and knockout of the TgBCKDH E1a subunit blocked production of acetyl‐CoA and led to disrupted in vitro and in vivo growth (Oppenheim et al., 2014). However, TgBCKDH E1a depletion did not result in any significant changes to BCAA levels, suggesting that mitochondrial acetyl‐CoA is not derived from BCAAs at least under normal growth conditions. In vitro enzymatic activity assays revealed that TgBCKDH has likely been repurposed to act as a PDH, which can be explained by sequence conservation of the catalytic residues between TgPDH E1a and TgBCKDH E1a (Oppenheim et al., 2014). This enzymatic repurposing serves as an example of the unique ways in which these parasites have diverged from their hosts.

Beyond producing energy in the form of ATP, the *T*. *gondii* mitochondrion also hosts critical biosynthetic pathways. As previously mentioned, one of the dehydrogenases that delivers electrons to the ETC, DHODH, plays a critical role in de novo pyrimidine biosynthesis by catalyzing the fourth step in the pathway (Hortua et al., 2012, 2016). Although *T*. *gondii* is capable of salvaging pyrimidines from the host, the salvage pathway does not seem to contribute significantly to pyrimidine levels in the parasite. Further, the de novo pathway has been shown to be critical for virulence both in vitro and in vivo (Fox & Bzik, 2002).

Another important biosynthetic pathway hosted by the *T*. *gondii* mitochondrion generates isoprenoids. Isoprenoids are a diverse group of molecules, including steroids, cholesterols, and ubiquinone, which play essential roles in membranes and ETC composition (Ling et al., 2007). Intracellular tachyzoites can likely salvage isoprenoids from the host cell while extracellular tachyzoites must synthesize their own (Li et al., 2013). Though isoprenoid precursors are generated in the apicoplast (Nair et al., 2011), *T*. *gondii* harbors a mitochondrial farnesyl‐diphosphate synthase (FPPS) that plays a key downstream role in isoprenoid synthesis (Ling et al., 2007). FPPS knockout disrupts mitochondrial membrane potential and ATP production, particularly in extracellular parasites (Li et al., 2013). Additionally, as mammalian hosts rely on a different pathway to produce isoprenoid precursors that can be blocked by statin drugs, combining statins with drugs that inhibit parasite isoprenoid production have been shown to work synergistically to cure mice of *T*. *gondii* infection (Li et al., 2013). As both classes of drugs are already approved for clinical use in humans, this combination calls for additional investigation as a potential treatment strategy.

Finally, the mitochondrion is also involved in the synthesis of two important iron‐containing molecules: heme and Fe‐S clusters. Heme molecules contain a central iron atom and serve as cofactors in a wide array of enzymes, notably those of the ETC (van Dooren et al., 2012). In mammals and yeast, heme biosynthesis is carried out between the cytosol and mitochondrion, while plant pathways also involve enzymes in the plastid. Interestingly, the localization of heme synthesis enzymes between the mitochondrion, cytosol, and apicoplast in apicomplexans combines aspects from both photosynthetic and non‐photosynthetic organisms (van Dooren et al., 2012). Of the eight enzymes involved in heme biosynthesis, the first and final two enzymes localize to the *T*. *gondii* mitochondrion (Bergmann et al., 2020). Heme biosynthesis was shown to be critical for maintaining levels of mitochondrial heme‐containing cytochrome proteins and for ATP production in *T*. *gondii* (Tjhin et al., 2020). Further, herbicidal compounds that inhibit the mitochondrial PPO (protoporphyrinogen oxidase), reduced heme abundance and *T*. *gondii* growth (Bergmann et al., 2020). As the *T*. *gondii* homolog of PPO is most closely related to plant PPO, the *T*. *gondii* heme biosynthetic pathway represents a potentially druggable target due to its divergence from that of the mammalian host (Bergmann et al., 2020). Similar in function to heme, Fe‐S clusters are another cofactor required for proper function of important proteins, particularly those involved in electron transport and metabolism (Lill, 2009). In plants, Fe‐S clusters are generated through three different pathways. The SUF (sulfur mobilization) pathway localizes to the plastid, the CIA pathway (cytosolic iron–sulfur cluster assembly) localizes to the cytosol, and the ISC (iron–sulfur cluster) assembly pathway localizes to the mitochondrion (Balk & Pilon, 2011). Plastids generally make Fe‐S for their own uses, while mitochondria contribute to general pools of Fe‐S (Balk & Pilon, 2011). As mammalian and yeast cells lack plastids, their Fe‐S clusters are synthesized exclusively via mitochondrial and cytosolic pathways (Ye & Rouault, 2010). Homologs of ISC, CIA, and SUF enzymes have been identified in apicomplexan genomes (Aw et al., 2021; Pamukcu et al., 2021). A homolog of a CIA enzyme, TgNBP35, was recently observed localizing to the OMM. While this enzyme localizes to the cytosol in most eukaryotes, it was shown to participate in cytosolic Fe‐S production in *T*.* gondii* and to play a critical role in parasite proliferation (Aw et al., 2021). Additionally, recent work showed that depletion of an enzyme involved in the mitochondrial ISC pathway of *T*.* gondii*, TgISU1, decreased expression of ETC proteins, disrupted mitochondrial membrane potential, and severely impacted parasite growth. Interestingly, depleting TgISU1 leads to initiation of an incomplete bradyzoite conversion in a type I strain, which typically does not form cysts (Pamukcu et al., 2021). This intriguing observation begs similar investigations in cystogenic type II strains. Our knowledge of Fe‐S synthesis in *T*. *gondii* remains fragmentary, and functional characterization of other enzymes in these critical pathways is still needed.

Importantly, much of what is known about *T*. *gondii* mitochondrial metabolism is based on studies in tachyzoites due to experimental constraints associated with studying slow‐replicating bradyzoites. Early enzymatic studies using cell extracts from purified bradyzoites illustrated that they had higher glycolytic enzyme and lactate dehydrogenase activities when compared to tachyzoite extracts. This suggested that bradyzoites may rely on energy production via glycolysis and lactate fermentation, as opposed to OXPHOS (Denton et al., 1996). Indeed, it was later shown that knockout of either *T*. *gondii* lactate dehydrogenase isoform (LDH1 or LDH2) leads to defects in bradyzoite differentiation or cyst burdens (Abdelbaset et al., 2017). Confusingly, some studies have shown that treating tachyzoites with ETC inhibitors induces in vitro expression of bradyzoite‐specific proteins (Tomavo & Boothroyd, 1995), while others have shown that ETC inhibitors are detrimental to bradyzoites in vivo (Ferguson et al., 1994). Thus, whether bradyzoites primarily rely on glycolysis, OXPHOS, or both for ATP production remains unclear. As there are currently no treatments against bradyzoites, further investigations into their metabolism could yield valuable therapeutic targets.

## BULLSEYE: THE MITOCHONDRION AS A DRUG TARGET

Although several biosynthetic pathways conserved throughout most eukaryotes are also present in the mitochondrion of the parasite, it is the high degree of divergent and essential biology in the organelle that makes it an attractive drug target (Alday & Doggett, 2017; Mather & Vaidya, 2008; Seidi et al., 2018). One of the major drug targets in apicomplexans is the ETC, where all complexes harbor apicomplexan‐specific subunits that could potentially be targeted by novel therapeutics (Maclean et al., 2021). As previously mentioned, the ETC plays a crucial role in energy production and is also involved in pyrimidine biosynthesis (Painter et al., 2007), making it a crucial element for parasite survival.

The first line of evidence showing that the mitochondrion could be a potential drug target came from the finding that a class of ETC complex III inhibitors, hydroxynaphthoquinone, were lethal to malaria parasites (Ellis, 1994). Atovaquone, which is currently used in combination with proguanil for malaria prophylaxis and treatment, binds to the Q_o_ site of parasite complex III (Mather et al., 2005; Srivastava & Vaidya, 1999). This binding effectively blocks CoQ oxidation, causing mitochondrial membrane potential collapse and inhibition of OXPHOS (Birth et al., 2014; Painter et al., 2007; Srivastava et al., 1997). Atovaquone is also used to treat *T*.* gondii* infections and is recommended for ocular toxoplasmosis as well as for patients who are not able to tolerate other drugs against this parasite (Dunay et al., 2018).

Aside from atovaquone, other related molecules such as endochin‐like quinolones (ELQs) also target complex III and are effective drugs against *T*. *gondii* and other apicomplexans (Doggett et al., 2012; Silva et al., 2020). ELQ316 targets the Qi site of complex III and ELQ400 seems to target both the Q_o_ and Q_i_ sites (Doggett et al., 2012; McConnell et al., 2018), making these compounds highly effective against apicomplexans. Mutations in the Q_o_ site are associated with atovaquone resistance (Akhoon et al., 2014; Vaidya & Mather, 2000), so any molecule targeting both complex III sites could mitigate the emergence of resistant parasites.

Another class of drugs, 1‐Hydroxyquinolone derivatives (HDQs), have been shown to inhibit the type II NADH dehydrogenases (NDH2) of *T*. *gondii* and *P*. *falciparum* (Saleh et al., 2007). Furthermore, a study revealed that these molecules also target dihydroorotate dehydrogenase (DHODH) and it is likely that the simultaneous inhibition of NDH2 and DHODH by HDQs impairs parasite growth (Hegewald et al., 2013). Finally, a recently published study demonstrated that the apicomplexan MQO, a mitochondrial enzyme with no orthologues in mammals, could represent a potential drug target (Acharjee et al., 2021). In *T*. *gondii*, MQO activity can be inhibited with ferulenol, and in *P*. *falciparum* inhibition is more pronounced in combination with atovaquone (Hartuti et al., 2018).

It is worth mentioning that most drug therapies against *T*. *gondii* focus on tachyzoites, the active, fast‐replicating stage of the parasite. Bradyzoites, the cyst‐forming stage of *T*. *gondii*, are impervious to current drug treatments (Alday & Doggett, 2017). There is evidence that atovaquone and ELQs can reduce tissue cyst burden in mice, suggesting that the ETC is required for bradyzoite survival and could be a potential drug target in this form of the parasite (Doggett et al., 2012). Although very little is known about mitochondrial function in bradyzoites, these findings suggest that developing therapies against the mitochondrion could be an effective way to target both tachyzoites and bradyzoites. Overall, targeting essential and divergent pathways in the *T*. *gondii* mitochondrion would leave its mammalian host unaffected. This organelle should, therefore, remain a prime target for therapeutic interventions.

## CONCLUDING REMARKS

From its highly fragmented and reduced mtDNA, to the protein complexes and enzymes essential for survival of the parasite, the mitochondrion of *T*. *gondii* provides unique opportunities to study apicomplexan‐specific biology and vulnerabilities. The role of numerous apicomplexan‐specific mitochondrial proteins remains to be elucidated, and we can be certain that studying them will yield insights into the unique functions they perform in these parasites. Moreover, since apicomplexans diverged from most model organisms ~400 million years ago, their mitochondrion could also shed light on the evolutionary pathway that this organelle took across different eukaryotic lineages.

In apicomplexans, the mitochondrion has been coevolving with another endosymbiotic‐derived organelle: the apicoplast. These organelles harbor essential biosynthetic and metabolic pathways, and, as exemplified by the heme biosynthetic pathway, they depend on each other for optimal functioning. Several studies have observed the mitochondrion and the apicoplast in close proximity (Kobayashi et al., 2007; Nishi et al., 2008). Although the significance of this proximity is currently unknown, it might facilitate the exchange of metabolites between the two organelles. In yeast and mammals, organelles in close apposition can interact with each other via membrane microdomains, termed membrane contact sites, that tether the two compartments together (Giacomello et al., 2020). With the recent discovery of an apicoplast two‐pore channel that regulates apicoplast‐ER membrane contact site formation in *T*.* gondii* (Li et al., 2021), it seems plausible that membrane contacts sites could also mediate the mitochondrion‐apicoplast interaction. While further work is necessary to confirm the presence of membrane contact sites between the mitochondrion and the apicoplast, identifying these structures could open new aspects of apicomplexan biology and yield insight into the evolution and molecular crosstalk of these organelles.

Historically, the mitochondrion of *T*. *gondii* was not only regarded as unremarkable, it was also overshadowed by the novelty and excitement surrounding the apicoplast. Nonetheless, with the advent of new sequencing, electron microscopy, and biochemical approaches, we now know that the mitochondrion is a hub of apicomplexan and myzozoan‐specific biology, and a compelling organelle in its own right. Numerous aspects of the *T*. *gondii* mitochondrion require further investigation. Among those, the mitoribosome composition and structure, and the mitochondrial metabolism of bradyzoites are some of the most pressing and exciting topics. Their study will undoubtedly generate valuable insights, and given that numerous mitochondrial proteins are absent from their mammalian hosts, the mitochondrion will certainly remain a prime drug target for novel therapeutics against *T*. *gondii* and other apicomplexans.

## Supporting information


**Table S1**. Gene names and IDs mentioned in this review.
