# Supplementary material for: Parasite powerhouse: A review of the Toxoplasma gondii mitochondrion
Source: J Eukaryot Microbiol. 2022 May 4;69(6):e12906. doi: 10.1111/jeu.12906 (PMC9490983; doi:10.1111/jeu.12906)
Supplement: Supplementary file 1 — Table S1. Gene names and IDs mentioned in this review. [file JEU-69-0-s001.pdf]

SUPPORTING INFORMATION

Parasite powerhouse: a review of the *Toxoplasma gondii* mitochondrion by

Madelaine M. Usey and Diego Huet

Table S1. Gene names and IDs mentioned in this review. Table compiling all *Toxoplasma gondii* gene names mentioned in this review, alongside their gene ID. For TgErv1, three different gene IDs have been reported (van Dooren et al. 2016).

Table S1. Gene names and IDs mentioned in this review. Table compiling all *Toxoplasma gondii* gene names mentioned in this review, alongside their gene ID. For TgErv1, three different gene IDs have been reported (van Dooren et al. 2016).

Gene Name	Gene ID
TgbL12m	TGME49_251950
TguL3m	TGME49_230050
TgmS35	TGME49_203620
TgTom40	TGME49_218280
TgTom22	TGME49_255245
TgSam50	TGME49_205570
TgTom7	TGME49_210255
TgTim22	TGME49_225710
TgTim23	TGME49_214150
TgTim17	TGME49_312220
TgTim50	TGME49_283590
TgTim44	TGME49_227830
TgmtHsp70	TGME49_251780
TgPam18	TGME49_202810
TgMPPα	TGME49_202680
TgMPPβ	TGME49_236210
TgErv	TGME49_210787, TGME49_288620, TGME49_232815
TgOxa1	TGME49_312430
TgNDH2-I	TGME49_288830
TgNDH2-II	TGME49_288830
TgMQO	TGME49_288500
TgG3PDH	TGME49_263730
TgDHODH	TGME49_210790
TgMDH	TGME49_318430
TgSDHA	TGME49_215590
TgSDHB	TGME49_215280
TgRieske	TGME49_320220
TgCytC1	TGME49_246540
TgQCR8	TGGT1_227910
TgQCR9	TGGT1_201880
TgQCR11	TGGT1_214250
TgQCR12	TGGT1_207170
TgApiCox25	TGGT1_264040
TgGAD	TGME49_280700
TgBCKDH E1a	TGME49_239490
TgPDH E1a	TGME49_245670
TgFPPs	TGME49_224490
TgPPO	TGGT1_272490
TgNBP35	TGME49_280730
TgISU1	TGGT1_237560
TgLDH1	TGME49_232350
TgLDH2	TGME49_291040
